# Early Initiation of Biologic Therapies to Prevent Severe Asthma Progression

**DOI:** 10.3390/medicina61101797

**Published:** 2025-10-06

**Authors:** Alessandra Tomasello, Alida Benfante, Stefania Principe, Nicola Scichilone

**Affiliations:** 1 Division of Respiratory Medicine, PROMISE Department, “Paolo Giaccone” University Hospital, University of Palermo, 90127 Palermo, Italy; alida.benfante@unipa.it; 2 Division of Allergy, Pulmonary and Critical Care Medicine, Vanderbilt University Medical Center, Nashville, TN 37232, USA; 3 Amsterdam UMC, Department of Pulmonary Medicine, University of Amsterdam, 1105 AZ Amsterdam, The Netherlands; s.p.principe@amsterdamumc.nl

**Keywords:** asthma, asthma remission, biologic therapy, biomarkers, airway remodeling, early intervention

## Abstract

Asthma is a chronic inflammatory disease with a heterogeneous course, often progressing silently from mild symptoms to severe, treatment-refractory disease. Current guidelines recommend biologic therapies after failure of high-dose inhaled corticosteroids and additional controllers, typically in patients with frequent exacerbations. This reactive approach may delay intervention until irreversible airway remodeling has occurred, limiting the potential benefits of biologic therapy. Therefore, severe asthma may be envisioned as the consequence of missed opportunities for early interventions. Early initiation of biologic therapy—guided by biomarkers such as blood eosinophil count and fractional exhaled nitric oxide (FeNO), as well as symptom burden and risk of lung function decline—may prevent progression to severe asthma and improve remission rates. This position paper advocates for a shift from severity-based to risk-based treatment strategies, recommending earlier biomarker assessment, redefinition of escalation criteria, and clinical trials designed to evaluate biologics in symptomatic non-exacerbating patients. By recognizing persistent inflammation and progression risk earlier in the disease course, clinicians may have a critical opportunity to alter the trajectory of asthma, reduce long-term morbidity, and achieve sustained control before irreversible damage occurs.

## 1. Introduction

Asthma is a heterogeneous chronic airway disease characterized by variable airflow obstruction, bronchial hyperresponsiveness, and chronic inflammation [1]. Clinical phenotypes range from infrequent mild symptoms to severe, refractory disease requiring high-dose inhaled corticosteroids (ICSs). Traditional asthma treatment paradigms focus primarily on symptom control and reducing acute exacerbations. However, this approach may overlook the progressive loss of lung function in patients with ongoing inflammation. Biologic therapies are monoclonal antibodies that target specific pathways of type 2 inflammation, such as interleukin (IL)-4, IL-5, IL-13, or immunoglobulin E (IgE), and are designed to reduce inflammation, improve asthma control, and minimize corticosteroid use in selected patients [2]. Current guidelines typically recommend biologic therapies only after failure of medium- or high-dose ICS and additional controllers [1]. This reactive strategy often delays intervention until substantial airway remodeling and steroid burden have occurred. Herein, we discuss the opportunities and challenges of how to position this new therapeutic alternative in practical clinical management and provide support to the proposal of a shift toward earlier initiation of biologics in appropriately selected patients, based not solely on exacerbation history, but also on biomarkers, symptoms, and risk of disease progression.

The domains of asthma risk include not only the short-term threat of exacerbations, but also the long-term risk of progression from mild to moderate to severe disease and systemic steroids exposure. While the current focus on severe asthma is justified, it is important to ask the following question: To what extent is severe asthma the consequence of inadequate early treatment and a failure to recognize progression risk in patients who initially present with “mild” disease? Guidelines recommend escalating ICS doses stepwise to maintain control [1]. For most patients, this results in years, often decades, of ICS exposure, particularly in those diagnosed in childhood when ICS treatment often begins by age 6, with long-term exposure continuing into adulthood. Historically, this approach was dictated by a lack of alternatives. Now, with effective and safe biologic therapies available, this strategy needs to be reevaluated. Asthma-related airway remodeling and progressive decline in lung function can begin early in the disease course [3,4,5]. Daily symptoms, even in the absence of frequent attacks, often reflect ongoing inflammation [6,7]. It is commonly accepted and supported by research findings that, if inadequately untreated, this chronic inflammation leads to fixed airway obstruction, tissue damage, and a lower likelihood of achieving clinical remission [8,9,10]. Biologic therapies offer a way to interrupt this trajectory [11]. It is logical to speculate that if used early, when the disease is still mainly sustained by an inflammatory and yet responsive state, they may prevent irreversible damage. This hypothesis is supported by findings that younger patients with less comorbidity and better preserved lung function are more likely to achieve remission on biologics [12,13,14]. Severe asthma is often a retrospective diagnosis, made only after years of steroid dependence, poor control, and lung function loss over time. This delay not only limits the effectiveness of biologics but also exposes patients to high-dose ICS and systemic corticosteroids, both of which are associated with adverse outcomes [15]. Medium to high doses of ICS have been linked to increased risks of cardiovascular events, pulmonary embolism, and pneumonia-related hospitalizations, particularly at higher doses [16,17]. We argue that the historical reliance on steroids, once necessary due to a lack of options, should now be reconsidered. This also applies to the inhaled formulations when given at high doses. We support the concept that patients who are dependent on high-dose ICS to control the disease should be evaluated early for biologic therapy, especially if they exhibit biomarkers of persistent inflammation.

The purpose of this review is to summarize current evidence, highlight specific treatment considerations, and identify key gaps in knowledge to guide future research and clinical practice. Although the present work is narrative in nature, a structured literature search was performed to enhance transparency. We searched PubMed for English-language articles published within the last 20 years using the keywords “asthma,” “biologic therapy,” “severe asthma,” and “early intervention” in the title or abstract fields. Reference lists of relevant publications were also manually screened to capture additional pertinent studies. Eligible articles included randomized controlled trials, systematic reviews, meta-analyses, and narrative reviews that addressed biologic therapies or risk-based treatment strategies in asthma.

## 2. Overview of Biologic Therapies in Asthma

The advent of biologic therapies has fundamentally transformed the management of asthma, offering precision-based strategies that directly target immunological drivers of disease. Currently, six biologics are approved by both the U.S. Food and Drug Administration and the European Medicines Agency: omalizumab, an anti-IgE antibody; mepolizumab and reslizumab, which neutralize IL-5; benralizumab, which induces eosinophil apoptosis via IL-5 receptor blockade; dupilumab, which inhibits IL-4 and IL-13 signaling by targeting the IL-4 receptor α; and tezepelumab, an antibody against thymic stromal lymphopoietin (TSLP). Collectively, these agents modulate key pathways of T2 inflammation, resulting in reduced exacerbation rates, improved lung function, enhanced quality of life, and decreased systemic corticosteroid use [18,19,20,21]. Omalizumab, the first biologic approved for asthma, mitigates allergen-induced inflammatory cascades by binding circulating IgE. Randomized controlled trials and systematic reviews consistently demonstrate that omalizumab reduces exacerbations and hospitalizations while providing modest gains in lung function and quality of life [22,23]. A 2006 Cochrane review of over 3000 patients with allergic asthma reported a 48% reduction in exacerbation risk and improved ability to taper ICS [24]. A subsequent 2011 meta-analysis confirmed these findings, showing a significant reduction in exacerbations and increased likelihood of stepping down ICS [25].

Mepolizumab has been extensively studied in severe eosinophilic asthma, with robust evidence demonstrating reduced exacerbations, improved asthma control, and significant oral corticosteroid-sparing effects [26,27,28,29,30]. Landmark trials such as DREAM [28], SIRIUS [26], and MUSCA [29] established its clinical efficacy, while real-world studies have illuminated its role in remission [31,32,33].

Reslizumab, like mepolizumab, targets IL-5 to reduce eosinophil-mediated inflammation. Administered intravenously, reslizumab has demonstrated significant reductions in exacerbation rates and improvements in lung function, particularly among patients with high baseline blood eosinophil counts (BECs) [22,34,35].

Benralizumab distinguishes itself from other IL-5 pathway agents by depleting eosinophils through antibody-dependent cell-mediated cytotoxicity. In phase 3 RCTs, benralizumab markedly reduced exacerbations, improved lung function, and enabled substantial reductions in oral corticosteroid use [36,37,38]. Long-term, open-label studies such as PONENTE [39] and ANDHI-In Practice [40] confirmed that clinical efficacy can be maintained even with step-down of background inhaled therapies.

Dupilumab, an IL-4 receptor α antagonist, inhibits both IL-4 and IL-13 signaling, central mediators of T2 inflammation. Clinical trials demonstrated substantial reductions in exacerbations, improved lung function, and pronounced oral corticosteroid-sparing effects [41,42]. Dupilumab is effective across allergic and eosinophilic asthma phenotypes, broadening its applicability beyond strictly eosinophilic disease. Moreover, improvements in biomarkers such as fractional exhaled nitric oxide (FeNO) and periostin highlight its capacity to attenuate airway inflammation across multiple T2 pathways. Real-world evidence indicates that dupilumab can induce multi-component remission, particularly in patients with high T2 biomarker expression, suggesting its potential role in early disease interception [43].

Tezepelumab, by targeting TSLP, exerts upstream effects across both T2-high and T2-low inflammatory pathways, representing the broadest-acting biologic to date. In the NAVIGATOR [44] and DESTINATION [45] trials, tezepelumab significantly reduced exacerbations and improved lung function regardless of baseline eosinophil count or allergic status. Importantly, clinical remission was achieved in nearly one-third of patients over two years, with higher success rates in those with preserved lung function and lower biomarker profiles at baseline. This ability to benefit a broad spectrum of severe asthma phenotypes makes tezepelumab particularly promising for early intervention aimed at preventing disease progression [46].

Together, the accumulated evidence underscores the efficacy, safety, and disease-modifying potential of biologics in severe asthma. Importantly, initiating biologics earlier in the disease course may maximize their potential not only to control symptoms but also to prevent irreversible airway remodeling and long-term disease progression. As asthma care advances toward a treat-to-target model, biologics are poised to play a central role in realizing remission and altering the natural history of severe asthma.

## 3. Evidence Supporting Early Use of Biologics

In recent studies of asthma remission, though definitions vary, the consistent trend is clear: the longer the disease duration, the lower the chance of remission with biologic therapy [14]. To quantify the proportion of adults with severe asthma achieving multidomain-defined remission after biologic initiation, and to identify prebiologic characteristics predictive of this outcome, a longitudinal cohort study was conducted using data from the International Severe Asthma Registry across 23 countries [14]. Four key outcome domains—symptom control, exacerbation frequency, lung function, and oral corticosteroid (OCS) use—were systematically assessed in the year before and after biologic initiation. Patients who subsequently achieved remission were characterized by less severe disease at baseline, evidenced by fewer exacerbations per year, lower daily maintenance OCS dose, better asthma control, and preserved lung function in the pre-biologic period. Shorter asthma duration was strongly associated with greater odds of remission, underscoring the importance of earlier initiation of biologic therapy. Other favorable baseline characteristics included younger age at treatment, lower body mass index, and absence or only low-dose use of maintenance OCS. Moreover, remission was more likely in patients with biological markers of type 2 inflammation, including higher BECs, elevated FeNO levels. Remission is more likely to occur in patients with better baseline lung function, fewer symptoms and exacerbations, minimal prior corticosteroid exposure, and higher BECs and FeNO levels [47,48,49]. These findings are supported by a retrospective analysis of severe asthma patients in the UK Severe Asthma Registry, which showed that the adjusted odds of remission were significantly lower in female patients (OR 0.61, 95% CI 0.45–0.93), obese individuals (OR 0.49, 95% CI 0.24–0.65), and those with uncontrolled symptoms (ACQ-5 ≥1.5; OR 0.19, 95% CI 0.12–0.31) prior to biologic therapy. Moreover, each additional 10 years of disease duration reduced the likelihood of remission by 14% (95% CI 0.76–0.97). Conversely, remission was more likely in patients with type 2–high biomarkers, shorter disease duration, and fewer comorbidities [49].

These findings reinforce the importance of early biologic intervention, before airway remodeling develops. In fact, remission rates in trials have ranged from 12 to 43%, mostly depending on timing of biologic initiation and patient characteristics [31,50,51,52]. Studies have shown that high BEC and FeNO levels are predictive of accelerated forced expiratory volume in one second (FEV_1_) decline, even in patients not experiencing frequent exacerbations [53,54,55]. In a large cohort of 4634 adults, serial assessments of BEC and FEV_1_ demonstrated that higher BECs were significantly associated with greater rates of lung function decline, independent of gender, height, and smoking status. Subgroup analyses revealed a dose–response effect: individuals with persistently elevated BECs experienced significantly greater FEV_1_ decline compared with those with consistently low BECs (<100/µL). Threshold effects were observed, whereby a BEC ≥100/µL in nonsmokers and ≥200/µL in smokers predicted accelerated FEV_1_ loss [53].

**Figure 1:**
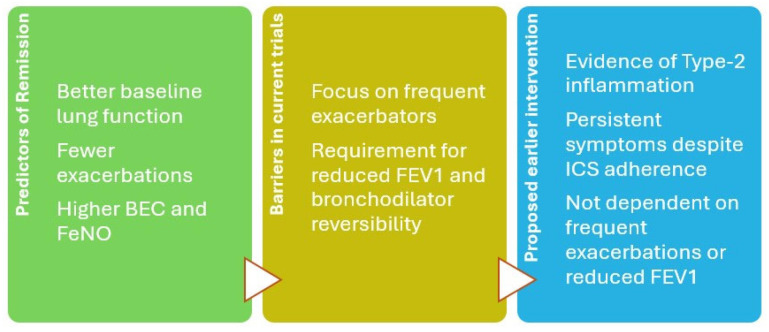
Diagram showing proposed earlier intervention.

These biomarkers offer a valuable opportunity to identify high-risk individuals at an earlier stage, enabling timely consideration of biologic therapies before fixed airflow limitation sets in. Improvements in lung function seen with biologic use are likely attributable to reduced airway smooth muscle constriction and edema, mediated by decreased eosinophilic infiltration into the airway submucosa and inhibition of chemotactic signaling. Most pivotal trials for biologics have focused on patients with frequent exacerbations (typically ≥2/year), reduced FEV_1_, and bronchodilator reversibility. However, bronchodilator reversibility is only present in ~15% of asthma patients [56], and many highly symptomatic patients are excluded from trials due to preserved lung function [57]. As a result, real-world patient profiles are underrepresented in the evidence base, and clinical guidelines reflect these narrow criteria. A proposed patient profile for earlier biologic intervention would therefore include patients with evidence of high type-2 inflammation (BEC ≥150 cells/µL and/or FeNO >25 ppb) and persistent symptoms despite adherence to inhaled therapy even in the absence of frequent exacerbations or reduced baseline FEV_1_ (Figure 1). To support earlier intervention, future trials should include symptomatic non-exacerbators, use lung function preservation and ICS-sparing as primary endpoints, and focus on the prevention of disease progression.

## 4. Proposed Revision of Clinical Practice and Research

**Figure 2:**
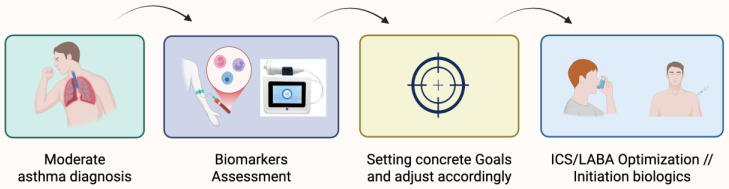
Proposed revised framework.

Patients with mild-to-moderate asthma who have persistently elevated BECs or FeNO, especially those experiencing daily symptoms or periodic attacks, should be identified as at high risk for progression. If conventional inhalation therapy fails to normalize biomarkers and control symptoms, treatment should be escalated to biologic agents earlier in the course of disease. For these patients, the traditional stepwise approach should be accelerated, with timely upward adding of biologics when inflammation remains active (Figure 2). Conversely, in patients with long-standing disease where inflammation has resolved and remodeling predominates, a different strategy may be needed, potentially focused on damage mitigation and non-biologic symptom management. Long-acting muscarinic antagonists, either as an add-on or in a triple inhaler with ICS-LABA, should be considered for patients with exacerbation history and lung function decline despite optimized therapy [58,59]. However, for earlier biologic intervention, the focus remains on patients with high type-2 inflammation and ongoing symptoms, even in the absence of frequent exacerbations or reduced baseline FEV_1_. This shift toward proactive intervention reflects an evolving view of asthma not only as a disease of exacerbations but also as a progressive disorder in which uncontrolled type 2 inflammation can silently accelerate lung function decline and promote structural remodeling. Biomarker-driven identification of these patients offers an opportunity for secondary prevention, potentially preserving long-term lung function and reducing cumulative corticosteroid exposure.

We recommend a proactive approach that includes the following: early biomarker assessment (blood eosinophils, FeNO, serum IgE) for all patients with moderate or symptomatic asthma, inclusion of symptom burden (even without frequent exacerbations), as a criterion for treatment escalation, escalation to biologic therapy if type 2 inflammation persists despite standard ICS/LABA therapy, regardless of exacerbation history. In clinical practice, this requires systematic biomarker monitoring at regular intervals and integration of results with symptom scores and lung function trends to guide treatment timing. Importantly, patients who exhibit persistent eosinophilia or elevated FeNO despite good adherence to inhaled therapy should be flagged as candidates for early biologic referral. On this basis, clinical trials could be re-designed to include symptomatic non-exacerbators and patients with early inflammation, define prevention-focused endpoints, such as time to lung function decline or steroid dependency, evaluate ICS-sparing effects and improvement in patient-reported outcomes as primary endpoints. Such trials would represent a paradigm shift toward disease modification, aligning asthma research more closely with approaches where prevention of irreversible organ damage is a primary goal. Finally, it is logical to speculate that, in specific conditions, the addition of a second biologic drug with a different modality of action would enhance the disease-modifying action.

## 5. Counterarguments and Responses

Some may raise concerns about the cost implications of introducing biologics earlier in the asthma treatment pathway. Indeed, biologic therapies are expensive, and healthcare systems may question whether extending their use by anticipating its introduction in asthma management is financially justifiable and affordable. In other words, sustainability committees could be forced to prioritize actions in real world medicine by choosing the most inexpensive strategy in short term scenario. However, when viewed through broader healthcare lens, early intervention with biologics may prove to be cost-saving. By preventing the accumulation of long-term damage, reducing hospitalizations, avoiding emergency care, and minimizing the need for systemic corticosteroids and high-dose ICS, each carrying substantial comorbidities and costs, biologics may ultimately reduce the economic and clinical burden of asthma. The question is then whether, and to what extent, asthma is considered a potentially debilitating and costly disease. The impression that asthma is perceived as a mild and therefore easily manageable disease both in the general population and in the decision-making authorities may lead to an underestimation of the overall impact of the consequences of this chronic respiratory disease to the patients and to their caregivers. A changing paradigm from the perception of an “occasional” to a “persistent” disease could raise the need to “hit early and hit hard” approach in order to prevent the progression and the worsening of this airway condition. To our opinion, this requires a capillary effort to disseminate knowledge at various levels in a broader manner. As discussed below, a proactive model that, rather than responding to acute worsening, aims at preventing it imposes courageous and firm actions.

Others may worry about the risk of overtreatment. While it is essential to avoid unnecessary escalation, this concern can be addressed through the early integration of objective biomarkers such as BEC and FeNO, as well as validated patient-reported symptom measures. These tools can help clinicians identify patients who are truly at risk of progression and ensure biologic therapy is used judiciously and only where appropriate. First, biomarkers of type-2 inflammation provide clear and reproducible thresholds for patient identification. BEC ≥ 150 cells/µL and/or FeNO >25 ppb are practical cut-offs supported by trial evidence and guideline recommendations (1). Higher thresholds (e.g., BEC ≥300 cells/µL or FeNO >50 ppb) strengthen the case for intervention, but even moderate elevations, when persistent, have been linked to accelerated lung function decline [52,53,54]. Second, symptoms and functional risk must be considered alongside biomarkers. Persistent symptoms despite optimized inhaled therapy (confirmed adherence, correct inhaler technique, and escalation to at least medium-to-high dose ICS) provide a clinical anchor to ensure that therapy is not escalated prematurely. Objective documentation of disease progression, such as accelerated decline in FEV_1_ (>30–40 mL/year) or a ≥10% relative drop from prior best [60,61], further strengthens the rationale for intervention. This combined approach, biomarker thresholds, symptom assessment, and objective evidence of progression, ensures that biologic therapy is used judiciously and directed toward the “right” patient at the right time, rather than indiscriminately. Moreover, evidence on follow-up and optimal treatment duration for patients receiving biologics at an earlier stage is currently lacking. In the absence of specific data, it is reasonable to follow current practice in severe asthma and continue biologic therapy while monitoring patient response, recognizing that stopping treatment may reduce effectiveness. Prospective studies are needed to establish clear guidelines for long-term management in this population.

Finally, there are safety considerations. It is worth emphasizing that biologic therapies approved for asthma have consistently demonstrated excellent safety profiles in both clinical trials and real-world use [62,63,64,65,66], including in pediatric and elderly populations [67,68]. Compared to the well-documented adverse effects of chronic corticosteroid use, such as osteoporosis, diabetes, cataracts, and cardiovascular events [69,70,71], biologics offer a favorable and more targeted alternative [26,38,68,72,73]. Thus, the concern should not be whether biologics are too risky to use early, but rather whether the risks of delaying them are too great to be ignored.

Current research efforts (e.g., NCT06676319) are beginning to explore new biologic therapies targeting key drivers of inflammation in patients with asthma who are not currently eligible for existing biologic treatments [74,75]. These studies aim to evaluate the potential of early intervention strategies in patients with mild-to-moderate disease who are considered at high-risk asthma.

## 6. Open Questions

Despite growing evidence supporting earlier biologic intervention, key knowledge gaps remain. First, there is no consensus on the optimal timing for initiating biologics in patients with moderate asthma and persistent type 2 inflammation. What constitutes a ‘high-risk’ patient in the absence of frequent exacerbations remains imprecisely defined. Second, the long-term outcomes of early biologic use, particularly in terms of preventing airway remodeling or altering disease trajectory, need to be established. Third, real-world identification and stratification of patients suitable for early biologic therapy pose practical challenges in many healthcare systems, including limited access to FeNO and eosinophil testing in a primary care setting. Lastly, cost-effectiveness models are needed to assess the broader health-economic impact of earlier intervention across diverse healthcare settings and populations. Addressing these questions through prospective, biomarker-stratified, real-world and randomized studies will be important to determine evidence-based guideline updates.

## 7. A Potential Shift Toward a Proactive Strategy

The evolving evidence not only focuses on earlier intervention, but also on the shift in asthma management philosophy, from a reactive, stepwise approach to a proactive model that goes beyond reacting to the worsening of symptoms or waiting for exacerbations. An emerging strategy emphasizes the proactive use of objective markers, such as blood eosinophil count, FeNO, and lung function trends, to guide treatment decisions and define therapeutic success. This goal-oriented approach shifts the focus from short-term symptom relief to measurable disease control and long-term preservation of lung function. Some authors have referred to this evolving paradigm as a “treat-to-target” strategy, in which therapy is adjusted until predefined goals, such as biomarker normalization, symptom resolution, or reduced steroid exposure, are achieved and maintained [76,77]. Initial studies suggest that biomarker-driven treatment adjustments can improve clinical outcomes, particularly when used to guide early escalation to biologic therapies in patients who show persistent type 2 inflammation despite standard inhaled treatment [76,77]. This approach aims to reduce the risk of irreversible airway remodeling and improve long-term disease control by introducing objective monitoring next to the regular clinical assessment. Although more prospective validation is needed, such a strategy aligns with broader trends in precision medicine and underscores the potential value of earlier, individualized intervention. When identifying patients who may benefit from earlier biologic intervention, it is essential to account for symptom triggers, comorbidities, and medication adherence. Optimizing environmental exposures, managing comorbid conditions such as obesity, allergic rhinitis, or gastroesophageal reflux, and confirming adherence to inhaled therapy help ensure that persistent symptoms reflect true disease activity, thereby allowing biologic therapy to be targeted to those most likely to benefit [1,78,79,80].

## 8. Conclusions

Severe asthma is too often a retrospective diagnosis, recognized only after years of inflammation and irreversible damage. What is still unknown is whether severe asthma is a mild disease that has worsened, or rather a different disease. In both cases, severe asthma may not be an inevitable destiny; it is often the consequence of missed opportunities for early intervention. Delaying biologic therapy until patients meet severe criteria reflects outdated trial constraints rather than the realities of disease biology. We now have the tools, biomarkers, targeted biologic therapies, and real-world data to intervene earlier and prevent progression in at-risk patients. We call for updated treatment pathways to allow for earlier biologic use, revise trial inclusion criteria to reflect real-world patient phenotypes, and support a shift from severity-based to risk-based treatment strategies. Asthma progression is not inevitable. Early identification and timely initiation of biologic therapy may offer the best chance to preserve lung function, reduce steroid burden, and achieve long-term remission. While the rationale behind the use of biologics and indirect evidence support early intervention, longitudinal clinical trials are needed to explore whether preemptive biologic therapy in non-severe, at-risk patients can prevent progression to severe disease.

When the fire is small, it is easier to extinguish it. In asthma, the same is true: the earlier we act, the greater our chance of preventing damage and achieving control that lasts.

## Data Availability

No new data were created or analyzed in this study.

## Abbreviations

BEC	blood eosinophil count
FeNO	Fraction exhaled nitric oxide
FEV_1_	Forced expiratory volume in one second
ICS	inhaled corticosteroids
IgE	Immunoglobulin E
IL	Interleukin
LABA	Long Acting Beta Agonist
T2	Type 2
TSLP	thymic stromal lymphopoietin

## References

[1] Global Initiative for Asthma (2025). Global Strategy for Asthma Management and Prevention, 2025. [Internet]. www.ginasthma.org.

[2] Backer V., Gibson P.G., Pavord I.D. (2023). The Asthmas: A Precision Medicine Approach to Treatable Traits, Diagnosis and Management.

[3] Winkler T. (2022). Mechanisms of airway remodeling converge at the critical point of bronchoconstriction in asthma. Ann. Transl. Med..

[4] Joseph C., Tatler A. (2022). Pathobiology of Airway Remodeling in Asthma: The Emerging Role of Integrins. J. Asthma Allergy.

[5] Pascual R.M., Peters S.P. (2005). Airway remodeling contributes to the progressive loss of lung function in asthma: An overview. J. Allergy Clin. Immunol..

[6] Miller R.L., Grayson M.H., Strothman K. (2021). Advances in asthma: New understandings of asthma’s natural history, risk factors, underlying mechanisms, and clinical management. J. Allergy Clin. Immunol..

[7] Cox J.K., Lockey R., Cardet J.C. (2025). Cough-Variant Asthma: A Review of Clinical Characteristics, Diagnosis, and Pathophysiology. J. Allergy Clin. Immunol. Pract..

[8] Tiddens H., Silverman M., Bush A. (2000). The Role of Inflammation in Airway Disease: Remodeling. Am. J. Respir. Crit. Care Med..

[9] Siddiqui S., Bachert C., Bjermer L., Buchheit K.M., Castro M., Qin Y., Rupani H., Sagara H., Howarth P., Taillé C. (2023). Eosinophils and tissue remodeling: Relevance to airway disease. J. Allergy Clin. Immunol..

[10] Pérez de Llano L., Marina Malanda N., Urrutia I., Martínez-Moragón E., Gullón-Blanco J.A., Díaz-Campos R., Esquerre M.M., Mena A.H., Cosío B.G., Cisneros C. (2023). Factors associated with suboptimal response to monoclonal antibodies in severe asthma. Allergy.

[11] Varricchi G., Poto R., Lommatzsch M., Brusselle G., Braido F., Virchow J.C., Canonica G.W. (2025). Biologics and airway remodeling in asthma: Early, late, and potential preventive effects. Allergy.

[12] Farinha I., Heaney L.G. (2024). Barriers to clinical remission in severe asthma. Respir. Res. [Internet].

[13] Thomas D., McDonald V.M., Pavord I.D., Gibson P.G. (2022). Asthma remission: What is it and how can it be achieved?. Eur. Respir. J..

[14] Perez-de-Llano L., Scelo G., Tran T.N., Le T.T., Fagerås M., Cosio B.G., Peters M., Pfeffer P.E., Al-Ahmad M., Al-Lehebi R.O. (2024). Exploring Definitions and Predictors of Severe Asthma Clinical Remission after Biologic Treatment in Adults. Am. J. Respir. Crit. Care Med..

[15] Sin D.D., Busse W.W. (2025). What Harm Are We Doing to Our Patients with Asthma by Using High-Dose Inhaled Corticosteroids?. Am. J. Respir. Crit. Care Med..

[16] Bloom C.I., Yang F., Hubbard R., Majeed A., Wedzicha J.A. (2025). Association of Dose of Inhaled Corticosteroids and Frequency of Adverse Events. Am. J. Respir. Crit. Care Med..

[17] Calverley P.M.A., Anderson J.A., Celli B., Ferguson G.T., Jenkins C., Jones P.W., Yates J.C., Vestbo J., TORCH investigators (2007). Salmeterol and Fluticasone Propionate and Survival in Chronic Obstructive Pulmonary Disease. N. Engl. J. Med..

[18] Brusselle G.G., Koppelman G.H. (2022). Biologic Therapies for Severe Asthma. Taichman DB, editor. N. Engl. J. Med..

[19] Sun B., Shen K., Zhao R., Li Y., Xiang M., Lin J. (2024). Precision medicine for severe asthma—Biological targeted therapy. Int. Immunopharmacol..

[20] Pelaia C., Crimi C., Vatrella A., Tinello C., Terracciano R., Pelaia G. (2020). Molecular Targets for Biological Therapies of Severe Asthma. Front. Immunol. [Internet].

[21] Lampalo M., Štajduhar A., Rnjak D., Ferara N., Stanić H.S., Popović-Grle S. (2025). Effectiveness of biological therapy in severe asthma: A retrospective real-world study. Croat. Med. J..

[22] Agache I., Beltran J., Akdis C., Akdis M., Canelo-Aybar C., Canonica G.W., Casale T., Chivato T., Corren J., Del Giacco S. (2020). Efficacy and safety of treatment with biologicals (benralizumab, dupilumab, mepolizumab, omalizumab and reslizumab) for severe eosinophilic asthma. A systematic review for the EAACI Guidelines—Ecommendations on the use of biologicals in severe asthma. Allergy.

[23] Hanania N.A., Alpan O., Hamilos D.L., Condemi J.J., Reyes-Rivera I., Zhu J., Casale T., Chivato T., Corren J., Del Giacco S. (2011). Omalizumab in Severe Allergic Asthma Inadequately Controlled With Standard Therapy: A Randomized Trial. Ann. Intern. Med..

[24] Walker S., Monteil M., Phelan K., Lasserson T.J., Walters E.H., The Cochrane Collaboration (2006). Anti-IgE for chronic asthma in adults and children. Cochrane Database of Systematic Reviews [Internet].

[25] Rodrigo G.J., Neffen H., Castro-Rodriguez J.A. (2011). Efficacy and Safety of Subcutaneous Omalizumab vs Placebo as Add-on Therapy to Corticosteroids for Children and Adults With Asthma. Chest.

[26] Bel E.H., Wenzel S.E., Thompson P.J., Prazma C.M., Keene O.N., Yancey S.W., Ortega H.G., Pavord I.D., SIRIUS Investigators (2014). Oral Glucocorticoid-Sparing Effect of Mepolizumab in Eosinophilic Asthma. N. Engl. J. Med..

[27] Ortega H.G., Liu M.C., Pavord I.D., Brusselle G.G., FitzGerald J.M., Chetta A., Humbert M., Katz L.E., Keene O.N., Yancey S.W. (2014). Mepolizumab Treatment in Patients with Severe Eosinophilic Asthma. N. Engl. J. Med..

[28] Pavord I.D., Korn S., Howarth P., Bleecker E.R., Buhl R., Keene O.N., Ortega H., Chanez P. (2012). Mepolizumab for severe eosinophilic asthma (DREAM): A multicentre, double-blind, placebo-controlled trial. Lancet.

[29] Chupp G.L., Bradford E.S., Albers F.C., Bratton D.J., Wang-Jairaj J., Nelsen L.M., Trevor J.L., Magnan A., Ten Brinke A. (2017). Efficacy of mepolizumab add-on therapy on health-related quality of life and markers of asthma control in severe eosinophilic asthma (MUSCA): A randomised, double-blind, placebo-controlled, parallel-group, multicentre, phase 3b trial. Lancet Respir. Med..

[30] Yancey S.W., Ortega H.G., Keene O.N., Mayer B., Gunsoy N.B., Brightling C.E., Bleecker E.R., Haldar P., Pavord I.D. (2017). Meta-analysis of asthma-related hospitalization in mepolizumab studies of severe eosinophilic asthma. J. Allergy Clin. Immunol..

[31] Thomas D., McDonald V.M., Stevens S., Harvey E.S., Baraket M., Bardin P., Bowden J.J., Bowler S., Chien J., Chung L.P. (2024). Biologics (mepolizumab and omalizumab) induced remission in severe asthma patients. Allergy.

[32] Pavord I., Gardiner F., Heaney L.G., Domingo C., Price R.G., Pullan A., Oppenheimer J., Brusselle G., Nagase H., Chupp G. (2023). Remission outcomes in severe eosinophilic asthma with mepolizumab therapy: Analysis of the REDES study. Front. Immunol..

[33] Crimi C., Nolasco S., Noto A., Maglio A., Quaranta V.N., Di Bona D., Scioscia G., Papia F., Caiaffa M.F., Calabrese C. (2024). Long-Term Clinical and Sustained REMIssion in Severe Eosinophilic Asthma Treated With Mepolizumab: The REMI-M Study. J. Allergy Clin. Immunol. Pract..

[34] Castro M., Zangrilli J., Wechsler M.E., Bateman E.D., Brusselle G.G., Bardin P., Murphy K., Maspero J.F., O’Brien C., Korn S. (2015). Reslizumab for inadequately controlled asthma with elevated blood eosinophil counts: Results from two multicentre, parallel, double-blind, randomised, placebo-controlled, phase 3 trials. Lancet Respir. Med..

[35] Deeks E.D., Brusselle G. (2017). Reslizumab in Eosinophilic Asthma: A Review. Drugs.

[36] Bleecker E.R., FitzGerald J.M., Chanez P., Papi A., Weinstein S.F., Barker P., Sproule S., Gilmartin G., Aurivillius M., Werkström V. (2016). Efficacy and safety of benralizumab for patients with severe asthma uncontrolled with high-dosage inhaled corticosteroids and long-acting β2-agonists (SIROCCO): A randomised, multicentre, placebo-controlled phase 3 trial. Lancet.

[37] FitzGerald J.M., Bleecker E.R., Nair P., Korn S., Ohta K., Lommatzsch M., Ferguson G.T., Busse W.W., Barker P., Sproule S. (2016). Benralizumab, an anti-interleukin-5 receptor α monoclonal antibody, as add-on treatment for patients with severe, uncontrolled, eosinophilic asthma (CALIMA): A randomised, double-blind, placebo-controlled phase 3 trial. Lancet.

[38] Nair P., Wenzel S., Rabe K.F., Bourdin A., Lugogo N.L., Kuna P., Barker P., Sproule S., Ponnarambil S., Goldman M. (2017). Oral Glucocorticoid–Sparing Effect of Benralizumab in Severe Asthma. N. Engl. J. Med..

[39] Menzies-Gow A., Gurnell M., Heaney L.G., Corren J., Bel E.H., Maspero J., Harrison T., Jackson D.J., Price D., Lugogo N. (2022). Oral corticosteroid elimination via a personalised reduction algorithm in adults with severe, eosinophilic asthma treated with benralizumab (PONENTE): A multicentre, open-label, single-arm study. Lancet Respir. Med..

[40] Louis R., Harrison T.W., Chanez P., Menzella F., Philteos G., Cosio B.G., Lugogo N.L., de Luiz G., Burden A., Adlington T. (2023). Severe Asthma Standard-of-Care Background Medication Reduction With Benralizumab: ANDHI in Practice Substudy. J. Allergy Clin. Immunol. Pract..

[41] Busse W.W., Maspero J.F., Rabe K.F., Papi A., Wenzel S.E., Ford L.B., Pavord I.D., Zhang B., Staudinger H., Pirozzi G. (2018). Liberty Asthma QUEST: Phase 3 Randomized, Double-Blind, Placebo-Controlled, Parallel-Group Study to Evaluate Dupilumab Efficacy/Safety in Patients with Uncontrolled, Moderate-to-Severe Asthma. Adv. Ther..

[42] Rabe K.F., Nair P., Brusselle G., Maspero J.F., Castro M., Sher L., Zhu H., Hamilton J.D., Swanson B.N., Khan A. (2018). Efficacy and Safety of Dupilumab in Glucocorticoid-Dependent Severe Asthma. N. Engl. J. Med..

[43] Pavord I.D., Rabe K.F., Israel E., Szefler S.J., Brusselle G., Pandit-Abid N., Altincatal A., Chen Z., Amin N., Khan A.H. (2025). Dupilumab Induces Long-Term On-Treatment Clinical Remission in Patients With Type 2 Asthma. J. Allergy Clin. Immunol. Pract..

[44] Corren J., Menzies-Gow A., Chupp G., Israel E., Korn S., Cook B., Ambrose C.S., Hellqvist Å., Roseti S.L., Molfino N.A. (2023). Efficacy of Tezepelumab in Severe, Uncontrolled Asthma: Pooled Analysis of the PATHWAY and NAVIGATOR Clinical Trials. Am. J. Respir. Crit. Care Med..

[45] Menzies-Gow A., Wechsler M.E., Brightling C.E., Korn S., Corren J., Israel E., Chupp G., Bednarczyk A., Ponnarambil S., Caveney S. (2023). Long-term safety and efficacy of tezepelumab in people with severe, uncontrolled asthma (DESTINATION): A randomised, placebo-controlled extension study. Lancet Respir. Med..

[46] Wechsler M.E., Brusselle G., Virchow J.C., Bourdin A., Kostikas K., Llanos J.P., Roseti S.L., Ambrose C.S., Hunter G., Jackson D.J. (2024). Clinical response and on-treatment clinical remission with tezepelumab in a broad population of patients with severe, uncontrolled asthma: Results over 2 years from the NAVIGATOR and DESTINATION studies. Eur. Respir. J..

[47] Oishi K., Hamada K., Murata Y., Matsuda K., Ohata S., Yamaji Y., Asami-Noyama M., Edakuni N., Kakugawa T., Hirano T. (2023). A Real-World Study of Achievement Rate and Predictive Factors of Clinical and Deep Remission to Biologics in Patients with Severe Asthma. J. Clin. Med..

[48] Lugogo N.L., Mohan A., Akuthota P., Couillard S., Rhoads S., Wechsler M.E. (2023). Are We Ready for Asthma Remission as a Clinical Outcome?. Chest.

[49] McDowell P.J., McDowell R., Busby J., Eastwood M.C., Patel P.H., Jackson D.J., Mansur A., Patel M., Burhan H., Doe S. (2023). Clinical remission in severe asthma with biologic therapy: An analysis from the UK Severe Asthma Registry. Eur. Respir. J..

[50] Milger K., Suhling H., Skowasch D., Holtdirk A., Kneidinger N., Behr J., Timmermann H., Schulz C., Schmidt O., Ehmann R. (2023). Response to Biologics and Clinical Remission in the Adult German Asthma Net Severe Asthma Registry Cohort. J. Allergy Clin. Immunol. Pract..

[51] Portacci A., Iorillo I., Quaranta V.N., Maselli L., Lulaj E., Buonamico E., Dragonieri S., Carpagnano G.E. (2023). Severe asthma clinical remission after biologic treatment with anti-IL4/IL13: A real-life experience. Respir. Med..

[52] Hansen S., Baastrup Søndergaard M., Von Bülow A., Bjerrum A.S., Schmid J., Rasmussen L.M., Johnsen C.R., Ingebrigtsen T., Håkansson K.E.J., Johansson S.L. (2024). Clinical Response and Remission in Patients With Severe Asthma Treated With Biologic Therapies. Chest.

[53] Lee S.H., Ahn K.M., Lee S.Y., Kim S.S., Park H.W. (2021). Blood Eosinophil Count as a Predictor of Lung Function Decline in Healthy Individuals. J. Allergy Clin. Immunol. Pract..

[54] Van Veen I.H., Ten Brinke A., Sterk P.J., Sont J.K., Gauw S.A., Rabe K.F., Bel E.H. (2008). Exhaled nitric oxide predicts lung function decline in difficult-to-treat asthma. Eur. Respir. J..

[55] Graff S., Demarche S., Henket M., Paulus V., Louis R., Schleich F. (2019). Increase in blood eosinophils during follow-up is associated with lung function decline in adult asthma. Respir. Med..

[56] Wang E., Wechsler M.E., Tran T.N., Heaney L.G., Jones R.C., Menzies-Gow A.N., Busby J., Jackson D.J., Pfeffer P.E., Rhee C.K. (2020). Characterization of Severe Asthma Worldwide. Chest.

[57] Lugogo N. (2025). The Rapidly Evolving Landscape of Asthma Therapies Is Exhilarating, Yet a Lack of Innovation in Clinical Trial Design Makes Feasibility A Real Concern. Am. J. Respir. Crit. Care Med..

[58] Sobieraj D.M., Baker W.L., Nguyen E., Weeda E.R., Coleman C.I., White C.M., Lazarus S.C., Blake K.V., Lang J.E. (2018). Association of Inhaled Corticosteroids and Long-Acting Muscarinic Antagonists With Asthma Control in Patients With Uncontrolled, Persistent Asthma: A Systematic Review and Meta-analysis. JAMA.

[59] Kew K.M., Dahri K. (2016). Long-Acting Muscarinic Antagonists (LAMA) Added to Combination Long-Acting Beta_2_-Agonists and Inhaled Corticosteroids (LABA/ICS) Versus LABA/ICS for Adults with Asthma. Cochrane Airways Group, Editor. Cochrane Database Syst. Rev. [Internet].

[60] Bucchieri S., Alfano P., Audino P., Cibella F., Fazio G., Marcantonio S., Cuttitta G. (2021). Lung Function Decline in Adult Asthmatics-A 10-Year Follow-Up Retrospective and Prospective Study. Diagnostics.

[61] Thomas E.T., Guppy M., Straus S.E., Bell K.J.L., Glasziou P. (2019). Rate of normal lung function decline in ageing adults: A systematic review of prospective cohort studies. BMJ Open..

[62] Kim S.H. (2024). Biologics in Severe Asthma: An Ideal Choice for Achieving Control. Allergy Asthma. Immunol. Res..

[63] Tat T.S., Cilli A. (2016). Evaluation of long-term safety and efficacy of omalizumab in elderly patients with uncontrolled allergic asthma. Ann. Allergy Asthma Immunol..

[64] Benson V., Vinals L., Freitag A., Sarri G., Day N., Alfonso-Cristancho R. (2024). long-term safety of biologics in asthma: A systematic literature review. Chest.

[65] Mohan A., Qiu A.Y., Lugogo N. (2024). Long-term safety, durability of response, cessation and switching of biologics. Curr. Opin. Pulm. Med..

[66] Scichilone N., Ventura M.T., Bonini M., Braido F., Bucca C., Caminati M., Del Giacco S., Heffler E., Lombardi C., Matucci A. (2015). Choosing wisely: Practical considerations on treatment efficacy and safety of asthma in the elderly. Clin. Mol. Allergy.

[67] Benfante A., Tomasello A., Gianquinto E., Cicero M.N., Scichilone N. (2023). Diagnostic and therapeutic approaches for elderly asthma patients: The importance of multidisciplinary and multidimensional management. Expert Rev. Respir. Med..

[68] Tomasello A., Benfante A., Lisotta A., Macaluso D., Viswanathan S., Cahill K.N., Scichilone N. (2024). Polypharmacy in older patients with asthma: Hidden risks and opportunities for improvement. Expert Rev. Respir. Med..

[69] Sullivan P.W., Ghushchyan V.H., Globe G., Schatz M. (2018). Oral corticosteroid exposure and adverse effects in asthmatic patients. J. Allergy Clin. Immunol..

[70] Price D.B., Trudo F., Voorham J., Xu X., Kerkhof M., Ling Zhi Jie J., Tran T.N. (2018). Adverse outcomes from initiation of systemic corticosteroids for asthma: Long-term observational study. J. Asthma Allergy.

[71] Waljee A.K., Rogers M.A.M., Lin P., Singal A.G., Stein J.D., Marks R.M., Ayanian J.Z., Nallamothu B.K. (2017). Short term use of oral corticosteroids and related harms among adults in the United States: Population based cohort study. BMJ.

[72] Bachert C., Han J.K., Desrosiers M., Hellings P.W., Amin N., Lee S.E., Mullol J., Greos L.S., Bosso J.V., Laidlaw T.M. (2019). Efficacy and safety of dupilumab in patients with severe chronic rhinosinusitis with nasal polyps (LIBERTY NP SINUS-24 and LIBERTY NP SINUS-52): Results from two multicentre, randomised, double-blind, placebo-controlled, parallel-group phase 3 trials. Lancet.

[73] Jackson D.J., Lugogo N., Gurnell M., Heaney L.G., Korn S., Brusselle G., Chanez P., del Olmo R., Llanos J.-P., Keeling N. (2025). Tezepelumab Reduces and Eliminates OCS Use in OCS-Dependent Patients With Severe Asthma: Primary Results From the Phase 3b WAYFINDER Study. Am. J. Respir. Crit. Care Med..

[74] Deiteren A., Krupka E., Bontinck L., Imberdis K., Conickx G., Bas S., Patel N., Staudinger H.W., Suratt B.T. (2025). A proof-of-mechanism trial in asthma with lunsekimig, a bispecific NANOBODY molecule. Eur. Respir. J..

[75] Tomasello A., Szefler S.J., Cahill K.N. (2025). Emerging systemic treatments for asthma and allergic diseases: New tricks, same dog?. J. Allergy Clin. Immunol. Pract..

[76] Pavord I.D., Beasley R., Agusti A., Anderson G.P., Bel E., Brusselle G., Cullinan P., Custovic A., Ducharme F.M., Fahy J.V. (2018). After asthma: Redefining airways diseases. Lancet.

[77] Busse W., Chupp G., Nagase H., Albers F.C., Doyle S., Shen Q., Bratton D.J., Gunsoy N.B. (2019). Anti-IL-5 treatments in patients with severe asthma by blood eosinophil thresholds: Indirect treatment comparison. J. Allergy Clin. Immunol..

[78] Gaffin J.M., Castro M., Bacharier L.B., Fuhlbrigge A.L. (2022). The Role of Comorbidities in Difficult-to-Control Asthma in Adults and Children. J. Allergy Clin. Immunol. Pract..

[79] Kaplan A., Szefler S.J., Halpin D.M.G. (2020). Impact of comorbid conditions on asthmatic adults and children. npj Prim Care Respir. Med..

[80] Maselli D.J., Sherratt J., Adams S.G. (2025). Comorbidities and multimorbidity in asthma. Curr. Opin. Pulm. Med..

